# IDH1^R132H^ Causes Resistance to HDAC Inhibitors by Increasing NANOG in Glioblastoma Cells

**DOI:** 10.3390/ijms20112679

**Published:** 2019-05-31

**Authors:** Geon-Hee Kim, So Young Choi, Taek-In Oh, Sang-Yeon Kan, Hyeji Kang, Sujin Lee, Taerim Oh, Hyun Myung Ko, Ji-Hong Lim

**Affiliations:** 1 Department of Applied Life Science, Graduate School of Konkuk University, College of Biomedical & Health Science, Konkuk University, Chungju 27478, Chungbuk, Korea; rlarjsgml4@kku.ac.kr (G.-H.K.); dk1050@kku.ac.kr (T.-I.O.); hsb6477@kku.ac.kr (S.-Y.K.); kkang@kku.ac.kr (H.K.); 201341532@kku.ac.kr (S.L.); 2 Diabetes and Bio-Research Center, Konkuk University, Chungju 27478, Chungbuk, Korea; 3 Department of Biomedical Chemistry, College of Biomedical & Health Science, Konkuk University, Chungju 27478, Chungbuk, Korea; sungjin30201@nate.com (S.Y.C.); dhxofla555@kku.ac.kr (T.O.); 4 Department of Life Science, College of Science and Technology, Woosuk University, 66 Daehak-ro, Jincheon-eup, Chungcheongbuk-do 27841, Korea; greatmen00@hanmail.com

**Keywords:** Glioblastoma, IDH1^R132H^, NANOG, HDACi, chemoresistance

## Abstract

The R132H mutation in isocitrate dehydrogenase 1 (IDH1^R132H^) is commonly observed and associated with better survival in glioblastoma multiforme (GBM), a malignant brain tumor. However, the functional role of IDH1^R132H^ as a molecular target for GBM treatment is not completely understood. In this study, we found that the overexpression of IDH1^R132H^ suppresses cell growth, cell cycle progression and motility in U87MG glioblastoma cells. Based on cell viability and apoptosis assays, we found that IDH1^R132H^-overexpressing U87MG and U373MG cells are resistant to the anti-cancer effect of histone deacetylase inhibitors (HDACi), such as trichostatin A (TSA), vorinostat (SAHA), and valproic acid. Octyl-(*R*)-2-hydroxyglutarate (Octyl-2HG), which is a membrane-permeable precursor form of the oncometabolite (*R*)-2-hydroxyglutarate (*R*-2HG) produced in IDH1-mutant tumor cells, significantly increased HDACi resistance in glioblastoma cells. Mechanistically, IDH1^R132H^ and Octyl-2HG enhanced the promoter activation of *NANOG* via increased H3K4-3Me, consequently increasing *NANOG* mRNA and protein expression. Indeed, HDACi resistance was attenuated in IDH1^R132H^-expressing glioblastoma cells by the suppression of NANOG using small interfering RNAs. Furthermore, we found that AGI-5198, a selective inhibitor of IDH1^R132H^, significantly attenuates HDACi resistance and NANOG expression IDH1^R132H^-expressing glioblastoma cells. These results suggested that IDH1^R132H^ is a potential molecular target for HDACi-based therapy for GBM.

## 1. Introduction

Isocitrate dehydrogenase 1 (IDH1) is a key metabolic enzyme for the conversion of citrate to α-ketoglutarate (α-KG) [1,2,3]. The most prevalent mutation in IDH1, R132H (IDH1^R132H^), was originally identified in acute myeloid leukemia (AML) and glioblastoma multiforme (GBM) [4,5,6]. However, the functional role of IDH1^R132H^ as a tumor suppressive, or oncogenic factor in tumor development and aggressiveness is debated [7,8,9,10,11,12,13,14]. A previous report has shown that conditionally overexpressed IDH1^R132H^ in hematopoietic lineages increases progenitor cell populations and extra-medullary hematopoiesis [15]. In an animal model, the knock-in of IDH1^R132H^ in adult mouse subventricular zone stem cells supports the oncogenic role of IDH1^R132H^ in gliomagenesis [10]. Paradoxically, increasing evidence has shown that the IDH1^R132H^ mutation suppresses glioma growth by the upregulation of microRNA-128a and downregulation of Wnt/β-catenin signaling [8,12,13]. Moreover, large-scale genomic studies of central nervous system tumors have shown that patients with IDH1^R132H^ have better clinical outcomes than those of patients with wild-type IDH1 [5,6]. 

IDH1^R132H^ gains neomorphic enzymatic activity for the synthesis of the R enantiomer of 2-hydroxyglutarate (*R*-2HG), which is structurally similar to α-KG [1,2,3]. Due to its similarity to α-KG, *R*-2HG alters the expression of genes related to cell differentiation and tumorigenesis by competitively inhibiting α-KG-dependent dioxygenases, such as TET family of 5-methylcytosine (5mC) hydroxylases and JmjC domain-containing histone demethylases (KDMs) [16]. Indeed, the suppressive effect of R-2HG on adipocyte differentiation in 3T3-L1 cells by inhibiting H3K9-specific demethylase KDM4C (Lysine Demethylase 4C) activity and downregulating adipogenic genes, such as CCAAT/enhancer-binding protein alpha (*CEBPA*), peroxisome proliferator-activated receptor gamma (*PPARG*), and adiponectin, has been observed [17]. Another report has shown that altered intracellular α-KG is sufficient to regulate pluripotency and self-renewal in human and mouse embryonic stem cells [18]. Thus, it is clear that intracellular α-KG, which is decreased by the IDH1^R132H^ mutation, fine-tunes cellular differentiation via α-KG-dependent dioxygenase-mediated transcriptional reprogramming.

Does IDH1^R132H^-driven *R*-2HG promote or suppress tumor development and growth in culture and in vivo? Koivenen et al. have shown that increased *R*-2HG in IDH1^R132H^-overexpressing human astrocytes enhances cellular proliferation and anchorage-independent growth by the activation of Egl nine homolog (EGLN) and inactivation of hypoxia-inducible factor (HIF) [19]. Consistent with these findings, several reports have shown that *R*-2HG promotes distant metastasis via epithelial-mesenchymal transition (EMT) in colorectal cancer and tumor progression via NF-kB activation in AML cells [20,21]. In contrast, a tumor suppressive effect of *R*-2HG in glioblastoma cells by the inhibition of ATP synthase and mTOR signaling has been reported [22]. Su et al. have shown that *R*-2HG exerts anti-leukemic activity by suppressing leukemia cell proliferation and cell cycle progression and increasing apoptosis [23].

Given that IDH1/2 mutations are early observed events in several cancers such as glioma, chondrosarcoma and AML, selective inhibitors targeting IDH 1/2 mutation are attractive strategies for cancer therapy [24]. For example, selective inhibitors, AGI-5198 and MRK-A, targeting IDH1^R132H^ have been identified by a high-throughput screen, and found to suppress production of *R*-2-hydroxyglutarate (*R*-2HG) and IDH1^R132H^-overexpressing glioma growth in a dose-dependent manner [25,26]. In addition, Enasidenib, which is an IDH2-mutant inhibitor, decreased production of R-2-hydroxyglutarate (*R*-2HG), and can be used for conventional therapy in AML. Nevertheless, many reports have shown that IDH1/2 mutant inhibitors could protect IDH1/2-muated glioma, chondrosarcoma, AML and colorectal carcinoma cells to multiple types of anti-cancer therapy, such as irradiation, daunorubicin, and PARP inhibitors [24,27,28,29]. For example, pretreatment of AGI-5198 attenuated anti-cancer efficacy of irradiation due to decreased 2-HG, restored NADPH production and decreased ROS levels [27]. Additionally, protective role of AGI-5198 against anti-cancer effect of poly (ADP-ribose) polymerase (PARP) inhibitor has been reported [28]. Based on these backgrounds, it is becoming clear that development of therapeutic application of IDH1/2 mutant inhibitors would be useful for treatment of IDH1/2-mutated cancer.

Increased predicted survival and better outcomes have been observed in low-grade glioma with temozolomide (TMZ), the most common anti-cancer drug used in patients with glioma [30,31]. Several reports have shown that glioma cells with IDH1^R132H^ are more sensitive than wild-type cells to radiotherapy and various chemotherapies, such as metformin and cisplatin [13,32,33,34]. It is becoming clear that histone deacetylase inhibitors (HDACi), such as trichostatin A (TSA), SAHA (also known vorinostat), and valproic acid, are promising anti-cancer drugs in multiple types of cancer [35]. Indeed, several pre-clinical studies of HDACs have shown that they target uncontrolled cellular proliferation, invasion, angiogenesis, and resistance to apoptosis in glioblastoma cells [36]. Based on these results from pre-clinical studies, vorinostat, the most advanced HDACi, has entered clinical trials in glioblastoma [36]. However, the IDH1^R132H^ mutation has not been evaluated for HDACi-based glioblastoma treatment.

The homeobox protein NANOG is an essential transcription factor that mainly regulates the self-renewal of embryonic stem cells and pluripotency [37]. NANOG regulates stem-like traits in cancer cells, such as proliferation, self-renewal, anchorage-independent growth, motility, epithelial-mesenchymal transition (EMT), immune evasion, and chemoresistance [38]. Indeed, several reports have shown that NANOG is abnormally increased and is closely associated with poor clinical outcomes in various types of cancer [39]. NANOG promotes multidrug resistance and immune evasion by increasing HDAC1; consequently, the inhibition of HDAC improves antigen-specific adoptive T-cell therapy as well as anti-cancer drug sensitivity [40]. In addition, suppressive effects of HDACi, such as TSA, vorinostat, and valproic acid, on the maintenance of cancer stem-like traits in pancreatic cancer cells and glioblastoma-derived stem cells have been reported [41]. 

Based on these previous results, we investigated whether IDH1^R132H^ overexpression alters the anti-cancer efficacy of HDACi in glioblastoma cells. In the present study, we showed that IDH1^R132H^ and its synthetic oncometabolite, R-2HG, significantly increase NANOG expression by activating its proximal promoter region, resulting in increased HDACi resistance. Furthermore, we found that the pharmacological and genetic suppression of IDH1^R132H^ and NANOG sufficiently attenuate HDACi resistance in IDH1^R132H^-expressing glioblastoma cells. Consequently, our results reveal that increased NANOG expression in glioblastoma with IDH1^R132H^ is a potential molecular target for therapeutic strategies.

## 2. Results and Discussion

### 2.1. Overexpression of IDH1^R132H^ Suppresses Viability, Motility, and Cell Cycle Progression in U87MG Glioblastoma Cells

**Figure 1:**
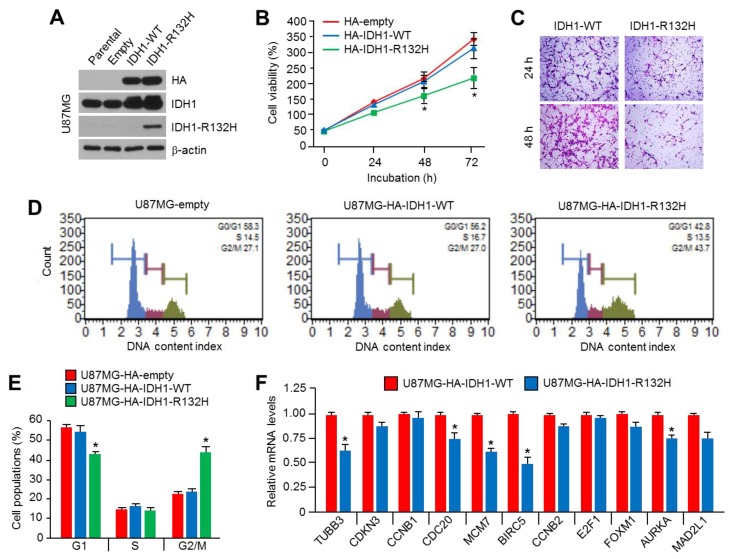
IDH1^R132H^ suppresses mitotic cell cycle and motility in U87MG glioblastoma cells. (**A**) Generation of IDH1^R132H^ overexpressing U87MG cells. IDH1^R132H^ overexpression was confirmed by using western blotting. (**B**) Overexpression of IDH1^R132H^ suppresses cell viability in U87MG cells. The stable cell lines were incubated for 24, 48, and 72 h, as indicated. Cell viability was measured by using crystal violet staining and assay. The values represent the mean ± SD of three independent experiments performed in triplicate; * *p* < 0.05. (**C**) Overexpression of IDH1^R132H^ suppresses cell motility in U87MG cells. Transwell chamber was used for in vitro cell migration assay. The stable cells were incubated for 24 or 48 h into upper chamber with MEM-α without fetal bovine serum (FBS) and 10% FBS containing culture medium was added into bottom chamber as chemotaxis. Decreased cell motility was shown as hematoxylin and eosin (H&E) stained images. (**D**) Overexpression of IDH1^R132H^ suppresses cell cycle at G2/M phase in U87MG cells. The stable cells were incubated for 24 h. Alteration of cell cycle progression was shown. (**E**) Quantitative cell population was shown. The values represent the mean ± SD of two independent experiments performed in triplicate; * *p* < 0.05. (**F**) Overexpression of IDH1^R132H^ suppresses cell cycle promoting genes expression in U87MG cells. The stable cells were incubated for 24 h, and then cell cycle promoting genes expression was measured by using qRT-PCR. The values represent the mean ± SD of three independent experiments performed in triplicate; * *p* < 0.05.

To determine whether the overexpression of IDH1^R132H^ has tumor suppressive or promoting effects in glioblastoma cells, we initially generated IDH1-wild type (WT) or IDH1^R132H^-mutant stably expressing U87MG cells. The overexpression of IDH1^R132H^ was confirmed (Figure 1A) and decreased cell viability in IDH1^R132H^-overexpressing U87MG cells was observed (Figure 1B). In addition, suppressed cell motility by IDH1^R132H^ overexpression was also observed (Figure 1C). To determine the mechanism by which IDH1^R132H^ overexpression suppresses cell viability in U87MG glioblastoma cells, we investigated the alteration of cell cycle progression. We found that the overexpression of IDH1^R132H^ increases the G2/M population by approximately 18% compared to those of the empty vector or IDH1-WT-overexpressing U87MG cells (Figure 1D,E). Indeed, decreased levels of genes that promote the cell cycle, such as Tubulin Beta 3 (*TUBB3*), cell-division cycle protein 20 (*CDC20*), minichromosome maintenance complex component 7 (*MCM7*), Baculoviral IAP Repeat Containing 5 (*BIRC5*), and Aurora Kinase A (*AURKA*), were observed in IDH1^R132H^-overexpressing U87MG cells (Figure 1F). Consistent with our results, previous work has shown that IDH1^R132H^ suppresses cell cycle progression and promotes apoptosis by inhibiting the Wnt/β-catenin signaling pathway [11]. These results suggest that IDH1^R132H^ acts as a tumor growth suppressive factor in U87MG glioblastoma cells.

### 2.2. Overexpression of IDH1^R132H^ Abolishes the Anti-Cancer Effect of HDAC Inhibitors

**Figure 2:**
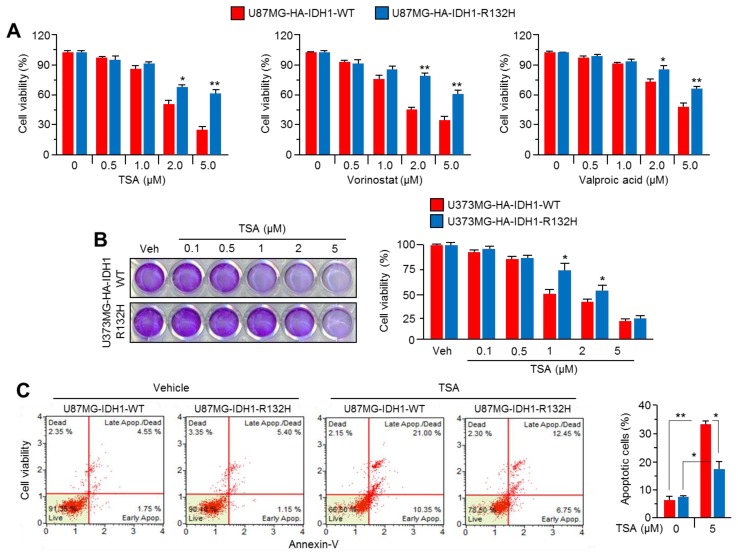
IDH1^R132H^ overexpression suppresses anti-cancer effect of HDAC inhibitors (HDACi) in glioblastoma cells. (**A**) IDH1-WT or IDH1^R132H^ overexpressing stable U87MG cells were incubated with TSA, vorinostat, or valproic acid for 48 h at various concentrations as indicated. The values represent the mean ± SD of three independent experiments performed in duplicate; * *p* < 0.05 and ** *p* < 0.01. (**B**) IDH1-WT or IDH1^R132H^ overexpressing stable U373MG cells were incubated with TSA for 48 h. Cell viability were measured by using crystal violet staining. Cell viability was measured by using crystal violet staining. The values represent the mean ± SD of three independent experiments performed in duplicate; * *p* < 0.05. (**C**) U87MG cells stably expressing IDH1-WT or IDH1^R132H^ were incubated with TSA for 72 h. Apoptotic cell population was measured by using Annexin-V staining. The values are presented as the mean ± SD of three independent experiments performed in duplicate; * *p* < 0.05 and ** *p* < 0.01.

Increasing data from preclinical and clinical studies of HDACi have shown that they are promising chemotherapeutics for the treatment of multiple types of cancer, including glioblastoma [42]; accordingly, we tested whether the overexpression of IDH1^R132H^ affects HDACi-based glioblastoma. Surprisingly, we found that the decreased cell viability by trichostatin A (TSA), vorinostat, or valproic acid, which are major Class I and II HDAC inhibitors, was significantly abolished in IDH1^R132H^-overexpressing U87MG glioblastoma cells (Figure 2A). In addition, the decreased anti-cancer effect of TSA was also observed in IDH1^R132H^-overexpressing U373MG cells (Figure 2B). To confirm this functional role of IDH1^R132H^ on HDACi resistance, the apoptotic cell population in the absence or presence of TSA was quantitatively analyzed in IDH1-WT or IDH1^R132H^-overexpressing U87MG cells. Figure 2C shows that the increased apoptotic cell population upon TSA treatment was significantly decreased by approximately 40% in IDH1^R132H^-overexpressing U87MG cells. These results revealed that the IDH1^R132H^ mutation might cause chemoresistance to HDACi-based glioblastoma therapy. 

### 2.3. NANOG Is Increased in IDH1^R132H^-Overexpressing U87MG and U373MG Glioblastoma Cells

**Figure 3:**
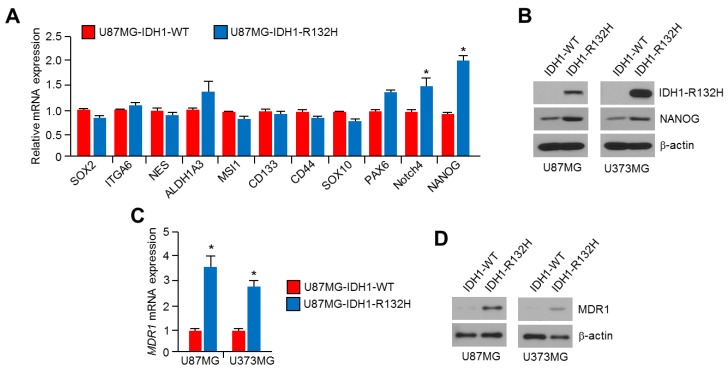
Overexpression of IDH1^R132H^ increases NANOG expression in U87MG and U373MG glioblastoma cells. (**A**) IDH1-WT or IDH1^R132H^ overexpressing U87MG cells were incubated for 24 h. Gene expression levels were measured by using qRT-PCR. The values represent the mean ± SD of three independent experiments performed in duplicate; * *p* < 0.05. (**B**) IDH1^R132H^ and NANOG protein levels in IDH1-WT or IDH1^R132H^ expressing U87MG and U373MG cells were measured by using Western blotting. (**C**) IDH1-WT or IDH1^R132H^ overexpressing U87MG cells were incubated for 24 h. Gene expression levels were measured by using qRT-PCR. The values represent the mean ± SD of three independent experiments performed in duplicate; * *p* < 0.05. (**D**) MDR1 protein levels in IDH1-WT or IDH1^R132H^ expressing U87MG and U373MG cells were measured by using Western blotting.

Increased gene expression, including the sex determining region Y-box 2 (*SOX2*), *CD133*, *CD44*, and *NANOG* in multiple types of cancer are closely associated with malignant phenotypes, such as angiogenesis, metastasis, and chemoresistance [38,43,44]. In addition, previous report has shown that embryonic stem (ES)-like gene signature, such as *NANOG*, *Oct4*, *SOX2* and *c-Myc*, was highly increased in grade 4 glioblastomas, which the most aggressive subtype of glioma, whereas lower or opposite ES-like signature was observed in lower grade glioblastoma and normal brain tissue [45]. Zbinden et al. also reported that NANOG modulates glioblastoma development and growth by acquisition of CD133+ stem cell behavior, gliomasphere clonogenicity and proliferation [46]. Thus, we further measured gene expression related to chemoresistance in IDH1^R132H^-overexpressing glioblastoma cells. We found that *NANOG* mRNA levels were predominantly increased by IDH1^R132H^ overexpression in U87MG cells (Figure 3A). In parallel with these findings, increased NANOG protein levels were also observed in IDH1^R132H^-expressing U87MG and U373MG glioblastoma cells (Figure 3B). 

Previous reports have shown that NANOG promotes chemoresistance by increasing multidrug resistance protein 1 (MDR1) also known as P-glycoprotein 1, which pumps anticancer agents out of the cells results in reduced intracellular drug concentration and cytotoxicity, expression [39,47]. Thus, we investigated whether increased NANOG by IDH1^R132H^ overexpression could functionally increase MDR1 expression. Increased *MDR1* mRNA and protein expression was observed in IDH1^R132H^ -overexpressing U87MG and U373MG cells (Figure 3C,D). These results suggest that NANOG and MDR1 levels are increased in IDH1^R132H^-expressing glioblastoma cells.

### 2.4. NANOG Promoter Is Activated with Increased H3K4-Trimethylation (H3K4-3Me) in IDH1^R132H^-Expressing U87MG Cells

**Figure 4:**
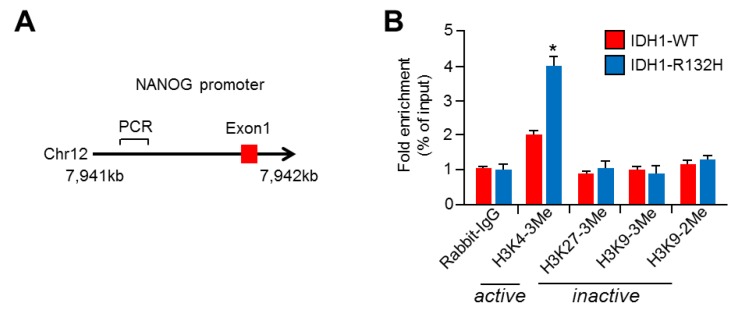
IDH1^R132H^ activates NANOG promoter with increased H3K4-3Me. (**A**) Schematic diagram of proximal region of NANOG promoter for ChIP-qPCR. Red box reveals exon 1 of NANOG gene and PCR primer sequence is provided in Materials section. (**B**) U87MG stable cell lines with IDH1-WT or IDH1^R132H^ expression were incubated for 24 h. Chromatin samples were incubated with antibodies as indicated. Purified DNA samples were subjected to qPCR to analyze the promoter region of NANOG gene. The values represent the mean ± SD of three independent experiments performed in duplicate; * *p* < 0.05. Fold changes were normalized to 1% of input sample.

IDH1^R132H^ regulates the expression of genes related to cellular differentiation by suppressing α-ketoglutarate (α-KG)-dependent dioxygenases, such as TET2 hydroxylase, histone demethylases, collagen prolyl 4 hydroxylases, and HIF prolyl 4 hydroxylases [48]. To understand how IDH1^R132H^ alters *NANOG* gene expression, we investigated epigenetic reprogramming on the proximal *NANOG* promoter region (Figure 4A). Interestingly, we found that H3K4-trimethylation (H3K4-3Me), a histone marker for active promoters, was significantly increased in the proximal *NANOG* promoter region, but H3K27-3Me, H3K9-3Me, and H3K9-dimethylation (H3K9-2Me) were not increased (Figure 4B). These results suggest that the *NANOG* promoter is activated in IDH1^R132H^-expressing U87MG cells. Consistent with our results, a previous ChIP-seq analysis has shown that increased R-2HG in IDH1^R132H^-expressing immortalized human astrocytes significantly promotes H3K4-3Me on the transcriptional start site of differentiation-antagonizing non-protein coding RNA (DANCR) and platelet-derived growth factor receptor A (PDGFRA) [49]. These results suggest that IDH1^R132H^ is sufficient to increase NANOG expression by activating its transcription with H3K4-3Me.

### 2.5. Knock-Down of NANOG Attenuates HDACi Resistance in IDH1^R132H^-Expressing U87MG and U373MG Glioblastoma Cells

**Figure 5:**
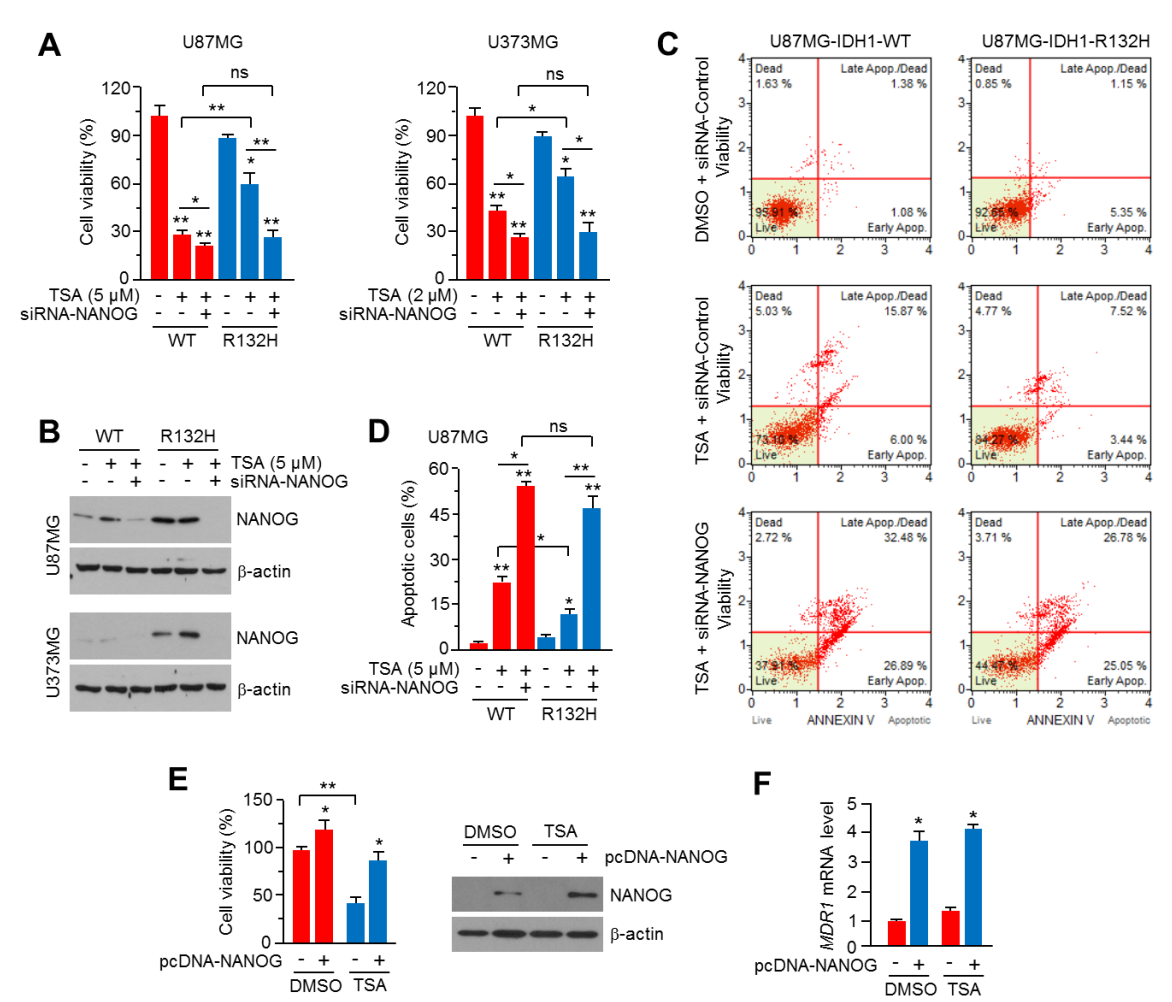
Suppression of NANOG abolishes TSA resistance in IDH1^R132H^ expressing U87MG and U373MG glioblastoma cells. (**A**) IDH1-WT or IDH1^R132H^ expressing U87MG or U373MG cells were transiently transfected with 20 nM of siRNA against control or NANOG. Transfected cells were incubated for 24 h to allow stabilization and further incubated with or without TSA for 48 h, as indicated. Cell viability was analyzed by using crystal violet staining. The values represent the mean ± SD of three independent experiments performed in duplicate; * *p* < 0.05 and ** *p* < 0.01. (**B**) Protein levels were measured by using western blotting. (**C**,**D**) IDH1-WT or IDH1^R132H^ expressing U87MG cells were transiently transfected with 20 nM of siRNA against control or NANOG. Transfected cells were incubated for 24 h to allow stabilization. After stabilization, cells were further incubated with or without TSA (5 μM) for 72 h. Apoptotic cell population was measured by using Annexin-V staining and Mini Flow Cytometry Muse™ Cell Analyzer. The values are presented as the mean ± SD of three independent experiments performed in duplicate; * *p* < 0.05 and ** *p* < 0.01. (**E**) U87MG cells were transiently transfected with pcDNA3.1-NANOG, and incubated for 24 h to allow stabilization. Transfected cells were incubated with or without 5 μM of TSA for 48 h. Cell viability was analyzed by using crystal violet staining and NANOG expression was measured by using western blotting. The values represent the mean ± SD of two independent experiments performed in triplicate; * *p* < 0.05 and ** *p* < 0.01. (**F**) *MDR1* mRNA levels were measured by using qRT-PCR. The values represent the mean ± SD of three independent experiments performed in duplicate; * *p* < 0.05.

Because increased NANOG is associated with chemoresistance to multiple types of anti-cancer drugs, including HDACi [40], we further investigated whether NANOG is closely linked to HDACi resistance caused by IDH1^R132H^ expression. Figure 5A shows that the knock-down of NANOG by small interfering RNA (siRNA) is sufficient to attenuate TSA resistance in IDH1^R132H^-expressing U87MG and U373MG glioblastoma cells. Indeed, a reduction of cell viability by approximately 50% in both IDH1^R132H^-overexpressing cells upon TSA treatment was observed (Figure 5A). In addition, strong knock-down of NANOG was also confirmed in both U87MG and U373MG cells (Figure 5B). Quantitation of the apoptotic cell population by annexin-V staining showed that the suppression of NANOG abolishes TSA resistance in IDH1^R132H^-expressing U87MG cells (Figure 5C,D). To confirm whether increased NANOG is functionally sufficient to cause TSA resistance in glioblastoma cells, cytotoxic effect of TSA was measured in NANOG overexpressed or parental U87MG cells. Increased cell viability upon both vehicle and TSA treatment was observed in NANOG overexpressed U87MG cells, suggesting that increased NANOG could be functionally associated with HDACi resistance (Figure 5E). In addition, increased *MDR1* mRNA levels were observed in NANOG-overexpressing U87MG cells, suggesting that increased MDR1 by NANOG could promote TSA resistance (Figure 5F). These results indicate that increased NANOG is critical for HDACi resistance in IDH1^R132H^-expressing glioblastoma cells.

### 2.6. Octyl-2-Hydroxyglutarate (Octyl-2HG) Increases NANOG and HDACi Resistance in Glioblastoma Cells

**Figure 6:**
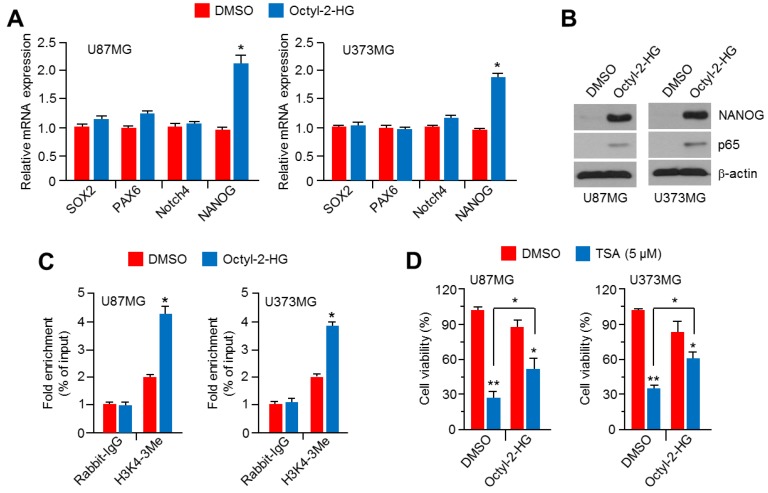
Octyl-2HG increases NANOG expression and causes TSA resistance in U87MG and U373MG glioblastoma cells. (**A**) U87MG and U373MG cells were incubated in the absence or presence of octyl-2HG (1 mM) for 24 h. Gene expression was measured by using qRT-PCR. The values represent the mean ± SD of three independent experiments performed in duplicate; * *p* < 0.05. (**B**) Protein levels were measured by using western blotting. (**C**) U87MG and U373MG cells were incubated with or without octyl-2HG (1 mM) for 24 h. Chromatin immunoprecipitation was performed by using antibody against for H3K4-3Me. Purified DNA samples were used to analyze the promoter region in NANOG gene. The values represent the mean ± SD of three independent experiments performed in duplicate; * *p* < 0.05. (**D**) U87MG and U373MG cells were incubated with or without TSA (5 μM) or octyl-2HG (1 mM) for 48 h. Cell viability was analyzed by using crystal violet staining. The values represent the mean ± SD of three independent experiments performed in duplicate; * *p* < 0.05 and ** *p* < 0.01.

The heterozygous mutation of IDH1 to IDH1^R132H^ produces R-2HG by disrupting the conversion of isocitrate to α-ketoglutarate [1,3]. Thus, we investigated whether R-2HG is sufficient to mimic IDH1^R132H^ overexpression to increase NANOG expression and HDACi resistance in glioblastoma cells. We found that cellular-permeable octyl-2HG is sufficient to increase NANOG mRNA and protein levels in U87MG and U373MG glioblastoma cells (Figure 6A,B). Consistent with previous results suggesting that NF-κB (p65) is increased by IDH1^R132H^ overexpression in AML cells [21], increased p65 levels were also observed in octyl-2HG-treated glioblastoma cells (Figure 6B). In addition, high enrichment for H3K4-3Me, a marker for active promoters, on the proximal *NANOG* promoter region was observed in octyl-2HG-treated U87MG and U373MG glioblastoma cells (Figure 6C). Surprisingly, we further found that octyl-2HG abolishes the anti-cancer effect of TSA in U87MG and U373MG cells (Figure 6D). These results reveal that R-2HG, as a crucial oncometabolite, might cause HDACi resistance in IDH1^R132H^-expressing glioblastoma cells. 

### 2.7. Pharmacological Inhibition of IDH1^R132H^ Attenuates HDACi Resistance in U87MG and U373MG Glioblastoma Cells

**Figure 7:**
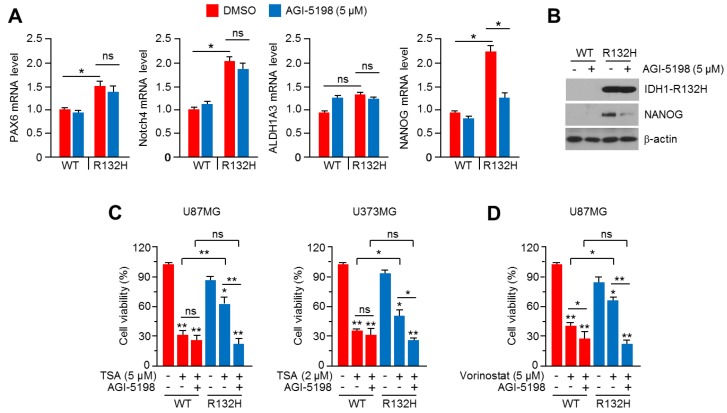
The beneficial effect of AGI-5198 on overcoming to HDACi resistance in IDH1^R132H^ overexpressing U87MG and U373MG glioblastoma cells. (**A**) IDH1-WT or IDH1^R132H^ expressing cells were incubated in the absence or presence of 5 μM of AGI-5198 for 24 h. Gene expression was measured by using qRT-PCR. The values represent the mean ± SD of three independent experiments performed in duplicate; * *p* < 0.05. (**B**) Protein levels were measured by using western blotting. (**C**) IDH1-WT or IDH1^R132H^ expressing U87MG or U373MG cells were incubated with or without TSA or AGI-5198 (5 μM) for 48 h, as indicated. Cell viability was analyzed by using crystal violet staining. The values represent the mean ± SD of three independent experiments performed in duplicate; * *p* < 0.05 and ** *p* < 0.01. (**D**) IDH1-WT or IDH1^R132H^ expressing U87MG cells were incubated with or without vorinostat (5 μM) or AGI-5198 (5 μM) for 48 h, as indicated. Cell viability was analyzed by using crystal violet staining. The values represent the mean ± SD of three independent experiments performed in duplicate; * *p* < 0.05 and ** *p* < 0.01.

A selective inhibitor of IDH1^R132H^, AGI-5198, with an anti-cancer effect in IDH1^R132H^-expressing glioma has been reported [25]. To provide a possible therapeutic strategy to overcome HDACi resistance in glioblastoma, we tested whether AGI-5198 is sufficient to attenuate HDACi resistance in IDH1^R132H^-overexpressing U87MG and U373MG cells. Initially, we found that AGI-5198 significantly decreases *NANOG*, but not *PAX6* or *Notch4*, in IDH1^R132H^-expressing U87MG cells (Figure 7A). Decreased NANOG protein levels were also observed in AGI-5198-treated IDH1^R132H^-expressing U87MG cells (Figure 7B). Figure 7C shows that AGI-5198 strongly suppresses TSA resistance by approximately 50% in IDH1^R132H^-overexpressing U87MG and U373MG glioblastoma cells. In addition, attenuated vorinostat resistance by AGI-5198 was also observed in IDH1^R132H^-expressing U87MG cells (Figure 7D). These results suggest that AGI-5198 is sufficient to abolish HDACi resistance in IDH1^R132H^-expressing glioblastoma cells. Given that selective inhibitors of IDH1/2 mutant could attenuate anti-cancer efficacy of irradiation, daunorubicin and PARP inhibitor [24], it is becoming clear that extended understanding of the therapy response-modulating effects of IDH1/2 mutations would provide useful therapeutic strategy for IDH1/2-mutated cancer treatment. In the present study, we found that a selective inhibitor, AGI-5198, efficiently overcame HDACi resistance in IDH1^R132H^-expressing glioblastoma cells, and our results provide that IDH1^R1332H^ targeting inhibitors, such as AGI-5198 and MRK-A, are promising strategy for improving the anti-cancer efficacy of HDACi-based treatment of IDH1^R132H^-mutated glioblastoma.

## 3. Materials and Methods

### 3.1. Reagents and Antibodies

Trichostatin A (TSA, T8552), Vorinostat (SML0061), Valproic acid (P4543), (2R)-Octyl-α-hydroxyglutarate (Octyl-2HG, SML2200) and AGI-5198 (SML0839) were purchased from Sigma Aldrich (St. Louis, MO, USA). Antibodies recognizing IDH1 (#3997), IDH1^R132H^ (SAB4200548), NANOG (sc-293121), p65 (sc-8008), and β-actin (sc-47778) were purchased from Cell Signaling Technology (Danvers, MA, USA), Sigma Aldrich (St. Louis, MO, USA), and Santa Cruz Biotechnology (Dallas, TX, USA). Antibodies for chromatin immunoprecipitation, H3K4-3me (ab8580), H3K27-3me (ab195477), H3K9-3me (ab8898), and H3K9-2me (ab32521) were purchased from Abcam (Cambridge, UK).

### 3.2. Cell Culture, Cell Viability Assay and Generation of Stable Cell Lines

Glioblastoma cell lines, U87MG and U373MG, were obtained from the Korean Cell Line Bank (Seoul, Korea). Cell lines were cultured in Minimum Essential Medium Eagle alpha medium (MEM-α) and Roswell Park Memorial Institute medium (RPMI1640) with 10% fetal bovine serum (FBS) and incubated in a humidified atmosphere at 37 °C under 20% O_2_ and 5% CO_2_. Cell viability was measured by using crystal violet staining [50]. HA-tagged IDH1-wild type (WT) or IDH1^R132H^ overexpressing U87MG and U373MG cells were seeded into 24-well tissue culture dishes and further incubated with or without HDAC inhibitors for 48 h. Then, cells were washed and fixed with phosphate-buffered saline (PBS) and 4% paraformaldehyde for 15 min at room temperature. After fixation, the cells were stained by using 0.5% crystal violet solution for 20 min, and then stained cells were lysed in 1% SDS solution. Optical density was obtained by using an absorbance reader at 570 nm (BioTek, Winooski, VT, USA) (OD570). 

### 3.3. Expression Constructs and Generation of Stable Cell Lines

HA-tagged IDH1-wild type (WT) expressing lentiviral vector (pLenti-GIII-CMV-IDH1-WT-HA; LV186028) was obtained from Applied Biological Materials (Richmond, BC, Canada). IDH1^R132H^ expressing lentiviral vector was generated in pLenti-GIII-CMV-C-term-HA by using the Quick Change point mutagenesis kit (Stratagene, La Jolla, CA) with the following primer sequence: 5’R132H (5’-ACCTATCATCATAGGTCATCATGCTTATGGG-3’) and 3’R132H (5’TGACCTATGATGATAGGTTTTACCCATCCAC-3’) [34]. pcDNA3.1-NANOG was a gift from Linzhao Cheng (Addgene plasmid # 28221) [51]. Lentiviruses encoding HA-tagged IDH1-WT or HA-tagged IDH1^R132H^ were produced in HEK293T cells with viral envelope and packaging vectors (pMD2G and psPAX2) using Lipofectamine (Invitrogen, Carlsbad, CA, USA), and amplified lentiviruses were collected 48 h post-transfection. To generate stably HA-tagged IDH1-WT or HA-tagged IDH1^R132H^ expressing U87MG and U373MG cells, lentiviral particles were added into both cells and incubated for 48 h, and then infected cells were selected with 2 μg/ml of puromycin.

### 3.4. Western Blotting

Protein samples were obtained from U87MG or U373MG cells using a protein extraction buffer [1% IGEPAL, 150 mM NaCl, 50 mM Tris-HCl (pH 7.9), 10 mM NaF, 0.1 mM EDTA, and a protease inhibitor cocktail], as previously described [50]. 40 μg of protein samples were subjected to sodium dodecyl sulfate (SDS)-polyacrylamide gel electrophoresis (PAGE) to separate protein by molecular weight, and then the separated proteins were transferred onto PVDF membranes (Millipore, Burlington, MA, USA). The transferred membranes were incubated with primary antibodies (1:1000) and then horseradish peroxidase (HRP)-conjugated secondary antibodies (1:10,000), respectively. Protein expression levels were visualized using an enhanced chemiluminescence (ECL) Prime kit (GE Healthcare, Pittsburgh, PA, USA).

### 3.5. Quantitative Real-Time PCR

Alteration of mRNA expression levels were measured by using Quantitative real-time PCR as previously described [50]. Total RNA was extracted by using TRIzol (Invitrogen, Carlsbad, CA, USA) and cDNA was synthesized by using a high-capacity cDNA reverse transcription kit (Applied Biosystems, Waltham, MA, USA). SYBR Green PCR Master Mix (Applied Biosystems, Waltham, MA, USA) was used for conducting quantitative PCR. The primer sequences for quantitative PCR are shown in Table 1.

### 3.6. Cell Cycle Analysis and Apoptosis Assays

Cell cycle analysis was performed using Muse™ cell cycle assay kit (Millipore, Burlington, MA, USA) and Mini Flow Cytometry Muse™ Cell Analyzer (Millipore, Burlington, MA, USA), as previously described [50]. Annexin-V staining was performed using Muse^TM^ Annexin V and Dead Cell Kit (Millipore, Burlington, MA, USA) to measure the apoptotic cells population [50]. HA-tagged IDH1-WT or IDH1^R132H^ overexpressing U87MG cells (1 × 10^5^ cells/well) were seeded onto a 6-well cell culture plate and further incubated with or without TSA for 72 h. Then, cells were washed and collected into fresh tubes. Collected cell pellets were mixed and stained with 100 μL Muse™ Annexin V and Dead Cell kit reagents (Millipore, Burlington, MA, USA) for 20 min at room temperature. Apoptotic cell numbers were analyzed by using Mini Flow Cytometry Muse™ Cell Analyzer (Millipore, Burlington, MA, USA).

### 3.7. Chromatin Immunoprecipitation (ChIP) Assay

HA-tagged-IDH1-WT or IDH1^R132H^ overexpressing U87MG cells were used for analyzing the epigenetic reprogramming on *NANOG* promoter region. The ChIP assay was performed by using the EZ-ChIP™ Chromatin immunoprecipitation kit (Millipore, Burlington, MA, USA). Briefly, cross-linked cells (1 × 10^7^) by using 37% formaldehyde were sonicated in SDS lysis buffer to shear chromatin to an average length of 200 bp to 1 kb. After chromatin shearing, 100 μL of samples were mixed with 900 μL of dilution buffer, and then diluted samples were incubated with protein A/G agarose beads and antibodies recognizing H3K4-3Me, H3K27-3Me, H3K9-3Me, or H3K9-2Me for overnight at 4°C. Immunocomplexes were washed using lithium chloride containing wash buffer, and then contaminated proteins and RNA were removed using proteinase K and RNase A. Finally, the purified DNA was used for measuring the enrichment of NANOG promoter region by using quantitative PCR. The primer sequences for ChIP-PCR are as follows: 5’-TGTGGCAGAAAGGATTGGA-3’ and 5’-TTGCAGGGTCATCATCAACG-3’ [52].

### 3.8. In Vitro Migration Assay

In vitro cell migration assay was performed using a Transwell chamber (Sigma-Aldrich, St. Louis, MO, USA) as previously described [53]. HA-tagged-IDH1-WT or IDH1^R132H^ overexpressing U87MG cells (3 × 10^3^) in 0.1 mL of MEM-α medium without fetal bovine serum (FBS) were seeded into the upper side of the Transwell chamber, and the lower side of the Transwell chamber was filled with MEM-α medium contained 6% FBS, and then cells were incubated for 12 h at 37 °C to allow migration from upper to lower side. After incubation, the Transwell insert membranes were washed using phosphate-buffered saline (PBS), and then migrated cells were stained using hematoxylin and eosin. The stained cells were captured using a microscope (Olympus, Tokyo, Japan).

### 3.9. Statistical Analysis

The unpaired Student’s t-test and one-way ANOVA with Tukey post hoc test were performed for statistical analysis, and the data are presented as the mean ± standard deviation (SD). A *p* value of < 0.05 was considered statistically significant.

## 4. Conclusions

The major findings of this study are that 1) IDH1^R132H^ suppresses glioblastoma cell growth and motility, 2) IDH1^R132H^ increases NANOG expression through activation of its proximal promoter region with increased H3K4-3Me, consequently causes resistance to HDACi, and 3) pharmacological inhibition of IDH1^R132H^ using AGI-5198 is sufficient to reduce NANOG expression and overcome HDACi resistance in IDH1^R132H^ expressing glioblastoma cells. Overall, our finding suggested that HDACi resistance by IDH1^R132H^-induced NANOG, which attenuated by IDH1^R132H^ inhibitor, AGI-5198, could be applied to improve therapeutic outcomes in glioblastoma patients with IDH1^R132H^ mutation.

## Figures and Tables

**Table 1:** Primer sequences for quantitative real time-PCR.

Gene	Forward Primer	Reverse Primer
TUBB3	AGCAAGAACAGCAGCTACTTCGT	GATGAAGGTGGAGGACATCTTGA
CDKN3	TCCAGTAGCTGCTTGTCTCCTACTATA	TCTTAGGTCTCGCAGGCTGTCT
CCNB1	AGCTGCTGCCTGGTGAAGAG	GCCATGTTGATCTTCGCCTTA
CDC20	GCCCACCAAGAAGGAACATC	TTTTCCACTGAGCCGAAGGA
MCM7	GGAAATATCCCTCGTAGTATCAC	CTGAGAGTAAACCCTGTACC
BIRC5	CGAGGCTGGCTTCATCCACT	ACGGCGCACTTTCTTCGCA
CCNB2	CCCAACTCCCTCTACCCTTGA	TCTGTCTCCCTCCCTCACTTTC
E2F1	CCCAACTCCCTCTACCCTTGA	TCTGTCTCCCTCCCTCACTTTC
FOXM1	TGCCCAGCAGTCTCTTACCT	CTACCCACCTTCTGGCAGTC
AURKA	GGAGAGCTTAAAATTGCAGATTTTG	GCTCCAGAGATCCACCTTCTCAT
MAD2L1	ACTTAAATATCTCCCTACCTATACTGAGTCAA	TAGTAACTGTAGATGGAAAAACTTGTGCTA
SOX2	CACATGAAGGAGCACCCGGATTAT	GTTCATGTGCGCGTAACTGTCCAT
ITGA6	GCTGGTTATAATCCTTCAATATCAATTGT	TTGGGCTCAGAACCTTGGTTT
NES	AGGCTGAGAACTCTCGCTTGC	GGTGCTGGTCCTCTGGTATCC
ALDH1A3	GCATGAGCCCATTGGTGTCT	CGCAGGCTTCAGGACCAT
MSI1	CTCCAAAACAATTGACCCTAAGGT	GACAGCCCCCCCACAAAG
CD133	AGAGCTTGCACCAACAAAGTACAC	AAGCACAGAGGGTCATTGAGAGA
CD44	TGCCGCTTTGCAGGTGTAT	GGCCTCCGTCCGAGAGA
SOX10	ACTTCGGCAACGTGGACATT	CAGCCACATCAAAGGTCTCCAT
PAX6	TTCAGAGCCCCATATTCGAG	GTTGGACACCTGCAGAAT
Notch4	AACTCCTCCCCAGGAATCTG	CCTCCATCCAGCAGAGGTT
NANOG	CCTCAGCCTCCAGCAGATGC	CCGCTTGCACTTCACCCTTTG
MDR1	TGACATTTATTCAAAGTTAAAAGCA	TAGACACTTTATGCAAACATTTCAA

## Abbreviations

IDH1	Isocitrate dehydrogenase
GBM	Glioblastoma multiforme
HDAC	Histone deacetylase
R-2HG	(R)-2-hydroxyglutarate
AML	Acute myeloid leukemia
5mC	5-methylcytosine
KDM4C	Lysine demethylase 4C
EMT	Epithelial-mesenchymal transition
α-KG	α-ketoglutarate
TMZ	Temozolomide
HIF	Hypoxia-inducible factor
